# Modeling Mode Choice Behavior Incorporating Household and Individual Sociodemographics and Travel Attributes Based on Rough Sets Theory

**DOI:** 10.1155/2014/560919

**Published:** 2014-11-06

**Authors:** Long Cheng, Xuewu Chen, Ming Wei, Jingxian Wu, Xianyao Hou

**Affiliations:** ^1^Jiangsu Key Laboratory of Urban ITS, Southeast University, Jiangsu Province Collaborative Innovation Center of Modern Urban Traffic Technologies, Si Pai Lou No. 2, Nanjing 210096, China; ^2^School of Transportation, Nantong University, Nantong 226019, China

## Abstract

Most traditional mode choice models are based on the principle of random utility maximization derived from econometric theory. Alternatively, mode choice modeling can be regarded as a pattern recognition problem reflected from the explanatory variables of determining the choices between alternatives. The paper applies the knowledge discovery technique of rough sets theory to model travel mode choices incorporating household and individual sociodemographics and travel information, and to identify the significance of each attribute. The study uses the detailed travel diary survey data of Changxing county which contains information on both household and individual travel behaviors for model estimation and evaluation. The knowledge is presented in the form of easily understood IF-THEN statements or rules which reveal how each attribute influences mode choice behavior. These rules are then used to predict travel mode choices from information held about previously unseen individuals and the classification performance is assessed. The rough sets model shows high robustness and good predictive ability. The most significant condition attributes identified to determine travel mode choices are gender, distance, household annual income, and occupation. Comparative evaluation with the MNL model also proves that the rough sets model gives superior prediction accuracy and coverage on travel mode choice modeling.

## 1. Introduction

Within the transportation field there exists many informative and detailed datasets that reveal a great deal about the travel behavior of households and individuals. However, it is the sheer volume and potential complexity of data that have discouraged these data from careful scrutiny.

Commonly used methods of travel mode choice modeling are based on the principle of random utility maximization derived from econometric theory. Since the multinomial logit (MNL) model [1] was developed in the 1970s, the parametric model family including different logit models with different structures and components has become the most widely used tool for mode choice analysis. However, many of these models suffer from the property of independence of irrelevant alternatives (IIA), which implies that the effects attributes of an alternative are compensatory and result in biased estimates and incorrect predictions in cases that violate the IIA property [2], although significant improvements on eliminating the IIA property have been made. Their predetermined structures may often misestimate or ignore partial relationships between explanatory variables and alternative choices for specific subgroups in a population. The linear property and synergy effects of the utility functions may not adequately model the comprehensive and complex correlations among explanatory variables and between them and dependent variables [3].

Another approach and the approach adopted in this paper are to embark on an exercise termed data mining or knowledge discovery, which makes few or no assumptions about the statistical nature of the data. Knowledge discovery can suggest the relationship between variables it contains using as few probability assumptions and linear structural relationships as possible. This information is usually contained in a series of rules that when they are evaluated to be true suggest a definite outcome. These rules can be expressed in the form of IF-THEN statements or in a tree-like structure. In this tree structure the internal nodes are decision tests; branches are paths from these decisions and terminal nodes are the outcome [4]. Other representations of the relationship between attributes in the data are also possible, including Bayesian networks [5] and neural networks [3]. In this paper, the knowledge is contained in the form of IF-THEN clauses. The technique for concluding these rules comes from the area of fuzzy set theory and in particular the rough sets application of this theory [6]. The characteristics of interest selected for the application of this theory are the travel mode choice of an individual for a trip.

Several recent studies of applying rough sets theory to travel behavior modeling [7–9] demonstrate the good benefits on prediction performance. However, existing researches mainly focus on long distance intercity travel analysis and few of them have compared the method with traditional MNL model. The primary objectives of this paper include (a) investigating the capability and performance on mode choice modeling of urban diary travel using rough sets theory, (b) figuring out the significance of condition attributes on mode choices, and (c) to comparatively evaluating the performance of rough sets model and MNL model.

## 2. Determinants of Travel Mode Choices

The most consistently quoted determinants of travel mode choices are individual demographics, including age, gender, education level, employment status, and availability of driver's license [10–14]. Young and elder individuals are more likely to utilize active modes of transportation. Women prefer to walk for active travel while men are more likely to utilize a bicycle. Individuals with higher levels of education walk significantly more than those with lower levels of education. Employed individuals are more likely to drive alone than unemployed individuals.

Other common determinants are the household characteristics, for example, income, household structure, and car and bicycle ownership [13, 15–17]. Households on higher incomes are more likely to own and use a car and families with children are more likely to use the car than one-person families. If households have cars, they would prefer to travel by cars. On the other hand, individuals with bicycle in their households have a higher propensity to participate in physically active pursuits.

Travel attributes could also impact people's mode choices [18]. When people go for work or school, they are more possibly to select motorized modes. Moreover, distance is an important factor for discrimination between modes of transport linked with higher costs (public transport and car/motorcycle) and those with lower costs (walking and cycling).

## 3. Data Source and Preparation

### 3.1. Travel Diary Survey

Data was collected from the activity-travel survey of Changxing County, China, in 2013. Changxing is a county in the prefecture-level city of Zhejiang Province, with the area of 42 km^2^ and the population of 250,000 residents. Taking a whole household as a unit, a random sampling and face-to-face interview were adopted for the survey on Wednesday, May 29, 2013. The investigators are required to select citizens randomly in different parts of the city in order to guarantee the quality of the sample. The sample involved a one-day (workday) activity-travel diary, which was designed to record all activities involving travel details such as purpose, mode, travel time, and origin destination of each trip, for all individuals above six years old in the household. It also included sociodemographics of both household and individual. Finally, 4831 valid forms from 1809 households were collected.

### 3.2. Data Preparation

The alternatives for travel mode choice used in this study are foot, bicycle (including tricycle), SOV (including moped and motorcycle), transit (including bus and company's vehicle), and car (including private car and taxi). When implementing rough sets analysis, returning purposes are excluded because mode choice of returning is largely associated with its former trip. This study is primarily concerned with the prediction of travel mode choice based on household and individual sociodemographics and travel attributes. These attributes and their corresponding categories are summarized in Table 1.

## 4. Rough Sets Theory

Rough sets theory is a mathematical framework that deals with vague and potentially conflicting data and was first formulated in the early 1980s [6]. The theory has been refined and developed into a powerful set of knowledge discovery and data mining techniques [19, 20] and is still an active area of research, with researchers working on the extensions of the theory [21, 22]. The theory has been implemented in a number of bespoke software such as ROSE [23], Rosetta [24], and RSES [25].

The theory belongs to the group of free-ranging algorithm and processes that aim to discover the knowledge contained within a dataset. In a dataset, it is possible to associate a particular outcome (e.g., travel mode choice) with a combination of values or levels held by other predictive attributes for a particular individual. When describing the process of deriving and applying the classification rules associated with rough sets, it is important to recognize that two stages are involved. Initially there is a training stage where there is an attempt to discover the knowledge and there then follows a testing stage where the predicative performance of this knowledge is tested.

### 4.1. Theory of Training

Let *U* represent the universe, a finite set of objects, and *A* denotes a set of condition attributes. For *x*, *y* ∈ *U*, we say that *x* and *y* are indiscernible by the set of condition attributes *A* if *ρ*(*x*, *q*) = *ρ*(*y*, *q*) for every *q* ∈ *A* where *ρ*(*x*, *q*) denotes the information function. A set that has objects within it that are indiscernible by the set of condition attributes *A* is called elementary set. The family of all elementary sets is denoted by *A* ^*^. It represents the smallest partitions of objects by the specified condition attributes so that objects belonging to different elementary sets are discernible and those belonging to the same elementary sets are indiscernible. The lower approximation of *X* (*X*⊆*U*), denoted by $\underline{A}X$, and the upper approximation of *X*, denoted by $\bar{A}X$, are defined as

(1) $$\begin{array}{c} \underline{A}X=\cup P\;\left\{P\in A^{*},P\subseteq X\right\},\\ \bar{A}X=\cup P\;\left\{P\in A^{*},P\cap X\neq \emptyset \right\}. \end{array}$$

**Figure 1:**
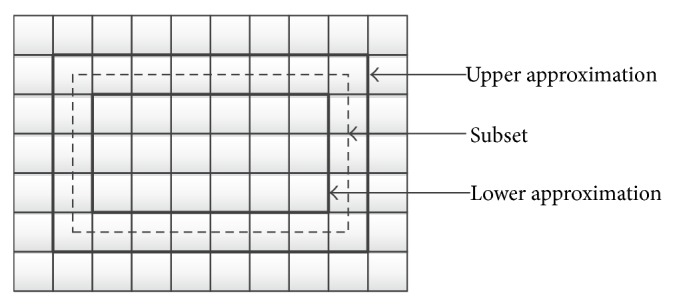
Approximation of sets.

The lower approximation contains all objects that certainly belong to that category. The upper approximation consists of all objects that possibly belong to that category. A rough set is thus any subset defined through its lower and upper approximation.  Figure 1 is a graphical representation of this concept. Each indiscernible set is displayed by a pixel. The subset of objects we want to approximate is drawn as a dashed line that crosses pixel boundaries and cannot be defined in a crisp manner. The lower and upper approximations are drawn as thick gridlines.

For example, five mode choice cases, described with four attributes, age, car ownership, purpose, and mode choice, are given in Table 2.

Mode choice case 1, for instance, is characterized by the following statement:* IF (age = young) AND (car ownership = yes) AND (purpose = work) THEN (mode choice = bus)*.

The above statement is called a rule in rough sets theory. The attributes in “THEN” part are called decision attribute which is the concept of concern, and attributes in “IF” part are called condition attributes which are the information we observe. The three condition attributes, age, car ownership, and purpose, form four elementary sets: {1,3}, {2}, {4}, {5}. It represents that cases 1 and 3 are indiscernible while other cases are characterized uniquely with condition attributes. Since cases 1 and 3 are indiscernible and lead to different mode choices, they are called boundary-line cases representing those that cannot be properly classified with the available information. Therefore, the bus mode choice is described with the lower approximation set, {2}, and the upper approximation set, {1,2, 3}. Similarly, the concept of car mode choice is characterized with its lower approximation set, {4,5}, and upper approximation set, {1,3, 4,5}.

Apparently, concepts can be described with different lower approximation and upper approximation by alternating input condition attributes. Sometimes, some particular condition attributes cannot be used to distinguish objects; they are redundant. The condition attributes excluding redundant attributes are called* reduct* in rough sets theory. A reduct is the essential part of an information table which can discern all objects discernible by the original table.

The performance of the specified condition attributes can be described with two indicators: accuracy of the approximation and quality of approximation. Accuracy of approximation represents the percentage of the associated objects definable with the specified condition attributes. It is defined as follows:

(2) $$\begin{array}{c} \alpha_{p}\left(X\right)=\frac{\text{card}\left(\underline{A}X\right)}{\text{card}\left(\bar{A}X\right)}, \end{array}$$

where cardrefers to cardinality. The value of accuracy ranges from 0 to 1. The closer to 1 is the accuracy, the more discernible is the condition attribute, that is, travel mode. It implies that the associated travel mode does exist unambiguously.

On the other hand, quality of approximation represents what percentage of the universe is definable. Let *X* = {*X*_1_, *X*_2_,…, *X*_*n*_} be a classification of *U*; that is to say, *X*_*i*_∩*X*_*j*_ = *∅*, ∀*i*, *j* ≤ *n*, *i* ≠ *j* and ⋃_*i*=1_^*n*^ *X*_*i*_ = *U*. *X*_*i*_ is called class of *X*. Quality of approximation of classification *X* by a set of attributes can be defined as follows:

(3) $$\begin{array}{c} \gamma_{p}\left(X\right)=\frac{\sum_{i=1}^{r}\text{card}\left(\underline{A}X_{i}\right)}{\text{card}\left(U\right)}. \end{array}$$

The value of quality ranges from 0 to 1. The closer to 1 is the quality, the more objects of the universe clearly belong to a single class of *X*. It implies that all travel modes can be clearly identified.

To recognize further details of mode choices,* rules* need to be extracted. Using reduced information table (without redundant attributes), the rules could be found through determining the decision attributes value based on condition attributes values. Therefore, the rules are presented in an “IF condition(s) THEN decision(s)” format. If the condition(s) in the IF part matches with the given fact(s), the decision(s) in the THEN part will be performed. Unlike mathematical functions or statistical models in traditional travel demand forecasting analysis, decision rules induced from a set of raw data can capture and represent both numeric and nonnumeric variables. In addition, the modular nature of decision rules makes it easy for researchers to insert new decisions rules or to modify/delete existing decision rules without affecting the overall system.

Once a set of rules have been derived, it is then that the training stage of the knowledge discovery finishes and the rules are then tested.

### 4.2. Theory of Testing

The testing stage is relatively straight forward and involves the application of rules to a previously unseen set of data in order to predict mode choice. Fortunately the actual mode choice is known so it is therefore possible to evaluate the predictive ability. This information is usually presented in a confusion matrix [26] which contains the actual mode choices as rows and the predicted mode choices as columns. The main diagonal is clearly the correct predictions and the off-diagonals are the incorrect predictions.

To evaluate the mode choice modeling performance of the rough sets, two prediction indicators are defined: accuracy of prediction and coverage of prediction. They, respectively, reflect the modeling performance on individual and aggregate level.

Accuracy of prediction (*γ*_*i*_) or hit ratio is the ratio of the number of correctly predicted individual observations for one mode (*N*_pi_) over the total number of the actual observations choosing this mode (*N*_*a*_), expressed as

(4) $$\begin{array}{c} r_{i}=\frac{N_{\text{pi}}}{N_{a}}. \end{array}$$

Coverage of prediction (*r*_*a*_) reflects the prediction accuracy on the mode aggregate level, defined as the ratio of the number of predicted observations (including correctly and incorrectly predicted observations) for one mode (*N*_pa_) over the number of the actual observations choosing this mode (*N*_*a*_), expressed as

(5) $$\begin{array}{c} r_{a}=\frac{N_{\text{pa}}}{N_{a}}. \end{array}$$

The accuracy is always less than 1 while the coverage may be greater than 1 or less than 1, with the accuracy rate being always no more than coverage rate. In the context of rough sets classification, accuracy alone is not a meaningful measure since the coverage affects how many classification attempts are made. Therefore, in this paper, accuracy and coverage are both utilized as the performance measures.

## 5. Applications to Travel Diary Survey

The software used to produce the results in this study is Rosetta [27]. In the application of knowledge discovery procedures to datasets, it is important that overfitting does not take place. This means that data used to derive the knowledge during the training stage are not the same as those used to test the knowledge. There are standard procedures to ensure that this does not take place. Where there is a limited amount of data, a *k*-fold procedure is adopted where the data is split into *k* mutually exclusive parts and then *k* training and testing procedures are conducted, but during each procedure one of the *k* parts is not used during the training stage but is held back for testing purposes. An alternative where there is sufficient data is to partition the data into two parts, one for exclusive training purposes and another for exclusive testing purposes. Since the travel data available in this study is large, it is this partition approach which has been adopted here. The data has been randomly split into two parts, 1/2 for the model estimation and another 1/2 for the subsequent validation test. The actual mode split proportions in the total database as well as the training set and testing set are shown in Table 3.

### 5.1. Approximation and Reduct

The accuracy of approximation is used to describe completeness of knowledge about decision attribute (travel mode) that could be obtained from condition attributes. As depicted in Table 4, foot shows the highest accuracy value of 91.9%. Other modes also have relatively good accuracy. This suggests that the twelve condition attributes (household and individual sociodemographics, travel attributes) could satisfactorily predict travel mode choices. On the other hand, quality of classification is the percentage of correctly classified cases. In this study, 91.9% of cases are correctly classified, indicating well-performed robustness of the rough sets model.

The reducts from the training set are calculated using the computationally efficient genetic algorithm option in Rosetta. The genetic algorithm is a heuristic for function optimization and promotes “survival of fittest” [28]. In total more than 3000 reducts are calculated. The length of the reducts is 2~12 attributes. It represents that any attribute is necessary for perfect approximation of the decision classes and removal of any of them leads to the decrease of the quality of approximation.

### 5.2. Decision Rule Induction

Based on the concepts of indiscernibility relations, set approximation, and attribute reduction, the training set is analyzed and over 40,000 rules are generated. This means that most rules are supported by just one or two objects. In fact, the highest support for an exact rule in this data is only 64 objects. The top five supported rules are shown in Table 5.

### 5.3. Validation

Confusion (or misclassification) matrix measures the effectiveness of the mode choice modeling.  Table 6 presents confusion matrix induced by the model for the testing set. In a confusion matrix, the sum on each row or column represents the actual or predicted number of observations for each mode. The main diagonal cells give the match number between reality and prediction and off-diagonal provides the erroneous classification. The accuracy and coverage for each mode appear in the table as the index of prediction performance.

Overall, the rough sets model has a good accuracy prediction, with the overall accuracy (hit ratio) up to 77.3%. The misclassification results reflect that it cannot distinguish between the SOV and car modes well in the fact that many observations under these two modes are mutually misclassified. This phenomenon indicates that the SOV and car modes, which share household, individual and travel attributes, exhibit more homogeneity within the explanatory variables than other modes. The model yields the highest prediction accuracy for foot with the rate up to 91.4%, showing most of the observations choosing the foot mode are not misclassified as other modes. However, the bicycle is underestimated heavily. A large part of the misclassified observations of the bicycle mode goes to the SOV mode, which may imply some unobserved similar preferences between SOV travelers and bicycle users.

On the other hand, the rough sets model made acceptable predictions of the mode choice distribution on the coverage level. It provides a relatively good coverage rate for the foot, SOV, and car modes but underestimates the aggregate numbers of observations of the bicycle and transit modes.

### 5.4. Significance of Condition Attributes

**Figure 2:**
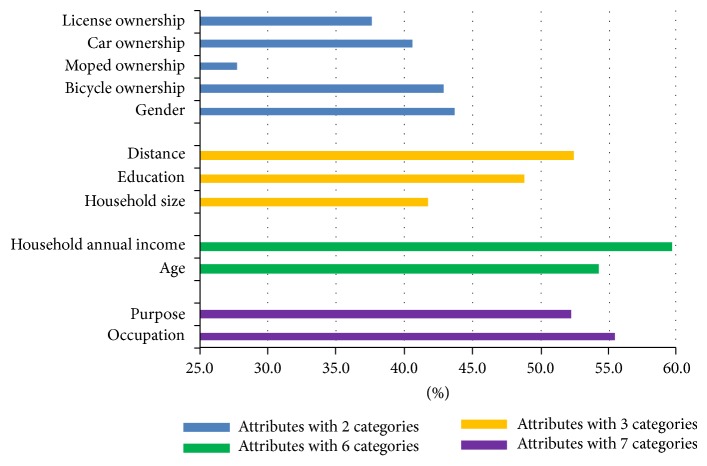
Presence percentage of condition attributes.

In rough sets models, the significance of condition attributes is measured by their presence of the derived rules [29]. When a condition attribute shows more frequently among rules, it is more frequently used to describe travel modes and hence more significant to distinguish mode choices. Presence of a condition attribute is represented with presence percentage which is calculated by summing its presence in each rule weighted with cases of the associated rule divided by total cases. Moreover, since condition attributes with more categories tend to distinguish between travel mode choices more effectively, comparisons are made on those with the same number of categories, shown in Figure 2.

There are total 12 condition attributes in this study selected to model mode choices. Figure 2 indicates that all variables make contributions to model estimation. Gender, distance, household annual income, and occupation are those with higher presence percentage among all condition attributes with two, three, six, and seven categories.

## 6. Comparisons with a Multinomial Logit (MNL) Model

The MNL model gives the choice probabilities of each alternative as a function of the systematic portion of the utility of all the alternatives. The general expression of the probability of choosing an alternative “*i*” from a set of *J* alternatives is as follows:

(6) $$\begin{array}{c} \mathrm{Pr}\left(i\right)=\frac{\exp\left(V_{i}\right)}{\sum_{j=1}^{J}\exp\left(V_{j}\right)}, \end{array}$$

where *Pr*⁡⁡(*i*) is the probability of the decision maker choosing alternative *i* and *V*_*j*_ is the systematic component of the utility of alternative *j*.

We use the same training set to estimate the MNL model. The car mode is arbitrarily used as the base alternative. From the estimation results, the most significant variables to influence a traveler's mode choice decision include car ownership, license ownership, gender, distance, and occupation. These variables approximately match the important variables induced by the rough sets models. The confusion matrix induced by the MNL model using the same testing set is shown in Table 7.

**Figure 3:**
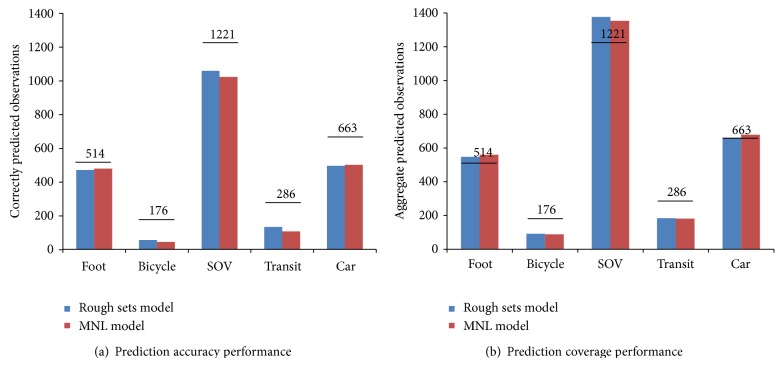
Prediction performance comparisons between rough sets model and MNL model.

An overall performance comparison was conducted based on the prediction results of the two models using the testing set.  Figure 3 shows the prediction accuracy and coverage of the models by each mode, in which the actual numbers of observations for each mode are also labeled.

The two models show similar prediction performances. Neither of them gives a perfect prediction rate for each mode on accuracy and coverage, especially for the insufficient observations in the dataset. On the accuracy of prediction, the rough sets model shows a better performance over the MNL model in the prediction of the bicycle, SOV, and transit modes. And the overall performance of the rough sets model (77.3%) is also better than the MNL model (75.2%).

On the prediction coverage, the MNL model shows better coverage on the SOV mode (110.8%) but performs worse on other modes. The rough sets model outperforms the prediction for the foot, bicycle, transit, and car modes. Another indicator, mean absolute percentage error (MAPE), was utilized to compare the coverage. MAPE is expressed as follows:

(7) $$\begin{array}{c} \text{MAPE}=\frac{\sum_{i=1}^{n}\left|{\text{PE}}_{i}\right|}{n},\\ {\text{PE}}_{i}=\frac{X_{i}-F_{i}}{X_{i}}, \end{array}$$

where PE_*i*_ is the prediction percentage error of observations for the *i*th travel mode, *X*_*i*_ is the actual number of observations for the *i*th mode, and *F*_*i*_ is the predicted number of observations for the *i*th mode.

The MAPE for rough sets model and MNL model is 20.6% and 21.7%, respectively. Thus, the rough sets model proves to be better on the overall prediction coverage.

## 7. Conclusions

This paper has demonstrated the successful application of a relatively new technique in the area of knowledge discovery to the well-studied problem of understanding and predicting traveler's mode choices. The method has been able to reveal information about the household characteristics, individual demographics, and travel attributes with mode choices in a readily understandable form (a set of “IF-THEN” statements) and to use this information to predict mode choice for previously unseen individuals.

The rough sets model shows high robustness of the model structure to the training dataset due to their data induction property. No statistical assumptions (e.g., IIA property assumption) need to be made so the compatibility between the model structure and the observations is enhanced in the model estimation and hence the prediction performance can be improved. According to presence of derived rules, the most significant condition attributes identified by the rough sets model of determining travel mode choices are gender, distance, household annual income, and occupation.

Comparative evaluation with the MNL model shows that the rough sets model has comparable but slightly better prediction capability on travel mode choice modeling. The prediction results based on separate testing dataset show, on both accuracy and coverage, that the rough sets mode outperforms the MNL model.

However, the rough sets induce too many detailed rules. Although the single rule is easy to interpret, the complete rule set is far too large to gain sound insight in travel behavior. Techniques such as generalization or shortening of the rule have been applied to deal with the problem [26]. Advanced models such as rough sets combined with genetic programming [30] can also be adopted in the future to improve the performance of rule extraction and observations validation.

## Figures and Tables

**Table 1:** Attributes and their descriptions.

Dimension	Attribute	Category
Household characteristics	Household size	1-2 persons; 3-4 persons; 5+ persons
	Household annual income	<30,000^*^; 30,000–60,000; 60,000–90,000; 90,000–120,000; 120,000–150,000; >150,000
	Car ownership	Yes; no
	Bicycle ownership	Yes; no
	Moped ownership	Yes; no

Individual characteristics	Gender	Male; female
	Occupation	Student; worker; employee; officer; businessman; retired; other
	Age	6~19; 20~29; 30~39; 40~49; 50~59; 60+
	Education	Middle school or below; high school; college
	License ownership	Yes; no

Travel attributes	Purpose	Work; school; business; leisure; bring/get someone; visit; other
	Distance	(0, 2 km]; (2, 5 km]; (5 km, +*∞*)
	Mode	Foot; bicycle; SOV; transit; car

Note: ^*^the unit is Yuan (RMB).

**Table 2:** Examples of mode choice cases with describing features.

Case	Age	Car ownership	Purpose	Mode choice
1	Young	Yes	Work	Bus
2	Old	No	Work	Bus
3	Young	Yes	Work	Car
4	Middle-aged	Yes	Work	Car
5	Middle-aged	No	Leisure	Car

**Table 3:** Summary of the mode splits in the datasets.

Mode	Total database		Training set		Testing set	
	Number	Per (%)	Number	Per (%)	Number	Per (%)
Foot	1022	17.9	508	17.8	514	18.0
Bicycle	384	6.7	208	7.3	176	6.2
SOV	2383	41.7	1162	40.6	1221	42.7
Transit	619	10.8	333	11.6	286	10.0
Car	1313	23.0	650	22.7	663	23.2

Sum	5721	100.0	2861	100.0	2860	100.0

**Table 4:** Approximation results.

Travel mode	No. of objects	Lower approx.	Upper approx.	Accuracy	Quality of classification
Foot	508	485	528	91.9%	91.9%
Bicycle	208	166	257	64.6%	
SOV	1162	1097	1248	87.9%	
Transit	333	282	387	72.9%	
Car	650	600	699	85.8%	

**Table 5:** Top supported induced decision rules.

Decision rules	Support
IF (bicycle ownership = no) AND (car ownership = yes) AND (gender = male) (occupation = businessman) AND (license ownership = yes) AND (distance = (5 km, +*∞*)) THEN (mode = car)	64

IF (bicycle ownership = no) AND (car ownership = no) AND (occupation = retired) AND (age = 60+) AND (purpose = leisure) AND (distance = (0,2 km]) THEN (mode = foot)	60

IF (bicycle ownership = no) AND (moped ownership = yes) AND (car ownership = no) AND (gender = female) AND (education = high school) AND (distance = (2,5 km]) THEN (mode = SOV)	59

IF (bicycle ownership = no) AND (car ownership = no) AND (household annual income = 60,000–90,000) AND (license ownership = no) AND (purpose = work) AND (distance = (2,5 km]) THEN (mode = SOV)	58

IF (household size = 1~2 persons) AND (bicycle ownership = no) AND (occupation = retired) AND (purpose = leisure) AND (distance = (0,2 km]) THEN (mode = foot)	58

**Table 6:** Confusion matrix generated by rough sets model.

	Testing set	Predicted mode choice					Accuracy (%)	Coverage (%)
		Foot (547)	Bicycle (92)	SOV (1376)	Transit (184)	Car (661)		
Actual mode choice	Foot (514)	**470**	7	27	2	8	91.4	106.4
	Bicycle (176)	39	**55**	70	4	8	31.3	52.3
	SOV (1221)	33	20	**1058**	20	90	86.7	112.7
	Transit (286)	3	5	85	**133**	60	46.5	64.3
	Car (663)	2	5	136	25	**495**	74.7	99.7

Overall accuracy (hit ratio)							77.3	

**Table 7:** Confusion matrix generated by MNL model.

	Testing set	Predicted mode choice					Accuracy (%)	Coverage (%)
		Foot (560)	Bicycle (88)	SOV (1353)	Transit (181)	Car (678)		
Actual mode choice	Foot (514)	**478**	6	29	0	1	93.0	108.9
	Bicycle (176)	42	**44**	77	8	5	25.0	50.0
	SOV (1221)	39	25	**1021**	31	105	83.6	110.8
	Transit (286)	1	7	106	**106**	66	37.1	63.3
	Car (663)	0	6	120	36	**501**	75.6	102.3

Overall accuracy (hit ratio)							75.2	

## References

[1] McFadden D., Zarembka P. (1974). Conditional logit analysis of qualitative choice behavior. *Frontiers in Econometrics*.

[2] Koppelman F. S., Wen C. H. (1998). Alternative nested logit models: structure, properties and estimation. *Transportation Research Part B: Methodological*.

[3] Xie C., Lu J., Parkany E. (2003). Work travel mode choice modeling with data mining: decision trees and neural networks. *Transportation Research Record: Journal of the Transportation Research Board*.

[4] Witten I. H., Frank E. (2005). *Data Mining: Practical Machine Learning Tools and Techniques*.

[5] Janssens D., Wets G., Brijs T., Vanhoof K., Timmermans H. Identifying behavioral principles underlying activity patterns by means of Bayesian networks.

[6] Pawlak Z. (1982). Rough sets. *International Journal of Computer and Information Sciences*.

[7] Goh C., Law R. (2003). Incorporating the rough sets theory into travel demand analysis. *Tourism Management*.

[8] Wang W., Namgung M. (2007). Knowledge discovery from the data of long distance travel mode choices based on rough set theory. *International Journal of Multimedia and Ubiquitous Engineering*.

[9] Witlox F., Tindemans H. (2004). The application of rough sets analysis in activity-based modelling. Opportunities and constraints. *Expert Systems with Applications*.

[10] Li Z., Wang W., Yang C., Jiang G. (2013). Exploring the causal relationship between bicycle choice and trip chain pattern. *Transport Policy*.

[11] Yang M., Li D., Wang W., Zhao J., Chen X. (2013). Modeling gender-based differences in mode choice considering time-use pattern: analysis of bicycle, public transit, and car use in suzhou, China. *Advances in Mechanical Engineering*.

[12] Xia X., Guan H. (2014). A study of the travel mode choice model of Chinese urban elderly. *Challenges and Advances in Sustainable Transportation Systems*.

[13] Bhat C. R., Srinivasan S. (2005). A multidimensional mixed ordered-response model for analyzing weekend activity participation. *Transportation Research B: Methodological*.

[14] Bhat C. R. (1998). Accommodating flexible substitution patterns in multi-dimensional choice modeling: formulation and application to travel mode and departure time choice. *Transportation Research Part B: Methodological*.

[15] Ryley T. (2006). Use of non-motorised modes and life stage in Edinburgh. *Journal of Transport Geography*.

[16] Dieleman F. M., Dijst M., Burghouwt G. (2002). Urban form and travel behaviour: micro-level household attributes and residential context. *Urban Studies*.

[17] Bhat R. C., Lockwood A. (2004). On distinguishing between physically active and physically passive episodes and between travel and activity episodes: an analysis of weekend recreational participation in the San Francisco Bay area. *Transportation Research A: Policy and Practice*.

[18] Müller S., Tscharaktschiew S., Haase K. (2008). Travel-to-school mode choice modelling and patterns of school choice in urban areas. *Journal of Transport Geography*.

[19] Pawlak Z. (1991). *Rough Sets: Theoretical Aspects of Reasoning about Data*.

[20] Pawlak Z. (2004). Some issues on rough sets. *Transactions on Rough Sets I*.

[21] Pawlak Z., Skowron A. (2007). Rudiments of rough sets. *Information Sciences*.

[22] Pawlak Z., Skowron A. (2007). Rough sets: some extensions. *Information Sciences*.

[23] Prędki B., Wilk S. (1999). Rough set based data exploration using ROSE system. *Foundations of Intelligent Systems*.

[24] Øhrn A., Komorowski J., Rosetta J. A rough set toolkit for analysis of data.

[25] Bazan J. G., Szczuka M. (2001). RSES and RSESlib—a collection of tools for rough set computations. *Rough Sets and Current Trends in Computing*.

[26] Clark S. D. (2009). Characterising and predicting car ownership using rough sets. *Transportation Research Part C: Emerging Technologies*.

[27] Øhrn A. (2001). *ROSETTA Technical Reference Manual*.

[28] Vinterbo S., Øhrn A. (2000). Minimal approximate hitting sets and rule templates. *International Journal of Approximate Reasoning*.

[29] Wong J. T., Chung Y. S. Using rough sets to explore the nature of occurrence of accidents.

[30] Hassan Y., Tazaki E. Rule extraction based on rough set theory combined with genetic programming and its application to medical data analysis.

